# A validation study: assessing the reliability of the hand held StatStripXPress lactate meter to test lactate in amniotic fluid

**DOI:** 10.1186/1756-0500-7-935

**Published:** 2014-12-19

**Authors:** Beverley Hall, Diana D Wong, William D Rawlinson, Mark B Tracy, Sally K Tracy

**Affiliations:** University of Sydney, Camperdown, Sydney, New South Wales 2006 Australia; Midwifery and Women’s Health Research Unit, Royal Hospital for Women, 1st Floor, Randwick, NSW 2031 Australia; Virology Research, Department of Microbiology, Prince of Wales Hospital, Randwick, NSW 2031 Australia; School of Medical Sciences, Faculty of Medicine, University of New South Wales, Kensington, NSW 2031 Australia; Department of Clinical Chemistry, South Eastern Area Laboratory Services, Randwick, 2031 Australia; Westmead NICU, Westmead Hospital, Westmead, NSW 2145 Australia

**Keywords:** Dystocia, Lactate, Amniotic fluid, Labour, Point of care

## Abstract

**Background:**

The level of lactate in amniotic fluid may provide useful clinical information when assessing whether a woman in labour is experiencing labour dystocia. If so, a rapid, reliable method to assess the concentration of amniotic fluid lactate at the bedside will be required in order to be clinically relevant. To assess efficacy, we compared the hand held StatStripXPreass lactate meter (Nova Biomedical) to the reference laboratory analyser ABX Pentra 400 (Horiba) in a controlled environment. Baseline biological lactate concentration was measured in triplicate and samples of a known quantity of thawed amniotic fluid spiked with lactate substrate (62 mmol/L) from the LDH12 kit (Roche, SUI) to yield a predetermined lactate concentration above baseline then measured in triplicate. Deming Regression was used to determine the linear agreement and a Bland Altman plot used to determine the paired agreement across the range of values.

**Findings:**

The mean difference with Bland-Altman plot between hand held meter and lab instrument was -1.0 mmol/L (SD 3.0 mmol/L) with 95% CI limits of agreement between -6.9 mmol/L to 4.9 mmol/L. The Deming regression co-efficient or slope of agreement was 0.91 (SD of 0.21).

**Conclusion:**

The measurement of amniotic fluid lactate using the StatStripXPress hand held meter was reliable compared to reference laboratory methods for measuring lactate levels in amniotic fluid.

## Rationale and background

Lack of progress in labour (dystocia) is one of the most commonly occurring problems requiring intervention during labour [1]. In many industrialized countries labours are treated for dystocia by augmenting with oxytocin to accelerate or improve progress [2, 3]. There is a need for improved diagnostic methods and decision making tools in the diagnosis of dystocia in order to reduce the high rates of labour intervention attributable to this condition.

The use of lactate as a measure of dystocia was first explored at the bedside by Wiberg-Itzel et al. [4]. This group found an association between higher concentrations of lactate in the amniotic fluid of women who were experiencing dysfunctional labour compared to those women labouring normally. They reported that labouring women with an amniotic fluid lactate level of >10.1 mmol/l were significantly more likely to require an operative delivery for labour dystocia [5]. The same group reported finding a correlation between poor neonatal outcomes and higher lactate levels in combination with other observational measures [6].

The studies by Wiberg-Itzel et al. [4, 5] are limited due to a lack of power to determine a strong association between the concentration of lactate in amniotic fluid and a resulting operative delivery. A further limitation was the use of a sophisticated pressure transducer to collect high samples of amniotic fluid during labour. This requires a level of operator skill that may not be available in resource poor settings or rural and remote maternity settings.

Our primary objective is to collect baseline measures of amniotic fluid lactate in a large sample population of nulliparous labouring women. We aim to determine the strength of the association between amniotic fluid lactate and dystocia using a hand held meter to test lactate concentration in amniotic fluid at the bedside. This small hand held device is portable, battery operated and able to process samples simply and quickly with minimum intrusion in the labour process. It has potential in rural and remote settings where technology may be out of reach because of cost and operator skill.

Although measurement of lactate using laboratory based spectrophotometric and fluorometric assays is possible, this is not clinically useful in diagnosing dystocia during labour due to the unavailability of such sophisticated equipment and time constraints in the clinical setting of active labour. In addition most advanced diagnostic laboratory technologies are centralised, and need highly trained staff and specialised facilities. The equipment is generally expensive and requires regular maintenance by skilled technicians.

If amniotic fluid lactate is relevant in the clinical management of dystocia, it is essential that a rapid and accurate test for amniotic fluid lactate is available at the bedside. To assess lactate in amniotic fluid we previously validated a handheld meter, the Lactate Pro (Arkray, Japan), approved in Australia for ‘sports’ use by the Australian Therapeutic Goods Administration Regulatory Agency (TGA) [7]. This meter is no longer approved by the TGA in our setting.

The StatStripXPress meter is currently approved by the TGA for clinical use therefore we aimed to assess the reliability of the StatStripXPress for use in our study of amniotic fluid lactate. Manufacturer’s specifications indicate the StatStripXPress meter measures lactate concentrations in whole blood between 0.3-20.0 mmol/L (2.7-180 mg/dL). We are not aware of any studies to validate the StatStripXPress for testing lactate in amniotic fluid.

The objective of this study was to determine the accuracy of the hand held lactate meter StatStripXPress (Nova Biomedical) compared to a reference laboratory analyser ABX Pentra 400 (Horiba) for the measurement of lactate in amniotic fluid.

## Study design and methods

### Ethics and consent

Written informed consent was obtained to collect amniotic fluid under sterile conditions from a woman with a singleton fetus undergoing elective amniocentesis in the third trimester of pregnancy. The Study Protocol was approved by New South Wales Northern Network Human Research Ethics Committee 11/140 through the National Ethics Application Form Submission AU/19E1A08.

### Validation of amniotic fluid lactate measurement

Baseline biological lactate concentration was measured in triplicate by testing the amniotic fluid samples with the StatStripXPress meter according to manufacturer’s instructions, prior to storing amniotic fluid at -80C. Briefly, a test strip was inserted into the StatXPress handheld meter, ensuring correct orientation. The device, with test strip in situ was dipped in a vertical position into the amniotic fluid sample. A numerical result was displayed in the meter window within 15 seconds and the result recorded. At the time of the validation study, amniotic fluid was thawed from -80C to room temperature and the baseline lactate level again tested in triplicate and recorded.

Samples of a known quantity of thawed amniotic fluid were spiked with lactate substrate (62 mmol/L) from the LDH12 kit (Roche, SUI) to yield a predetermined lactate concentration above baseline. The selected values were based on previous lactate studies [7, 8]. Samples were spiked with lactate and mixed by pulse-vortex before determination of lactic acid concentration using the handheld meter or the ABX Pentra 400. For the handheld meter, ~5 μl of the sample was applied onto a clean plastic surface for uptake in test strips in triplicates. At the time of spiking, samples were first tested using the StatStripXPress meter and within one hour of spiking, formal analysis of lactate levels was performed using the ABX Pentra 400 (Horiba) (Table 1).

**Table 1:** **A comparison of lactic acid concentrations detected between StatStripXPress handheld meter and automated lactate analyser (Abx Pentra 400) results for amniotic fluid collected under sterile conditions and spiked with a known quantity of lactate**

	StatStripXPress		AbX Pentra 400	
Concentration (mmol/L)	Concentration + baseline (mmol/L)	Mean ± SD ^§^ (mmol/L)	Concentration + baseline (mmol/L)	mmol/L
Baseline (pre-freeze)		9.1 ± 0 .40	Not tested	
Baseline (post-thaw)		8.6 ± 0.60	7.70	7.7
5	13.6	11.6 ± 1.24	12.7	12.2
6	14.6	13.9 ± 1.86	13.7	13.8
7	15.6	15.6 ± 2.27	14.7	14.1
8	16.6	13.2 ± 4.76	15.7	15.0
9	17.6	15.1 ± 1.73	16.7	16.0
10	18.6	16.5 ± 1.58	17.7	16.8
11	19.6	17.1 ± 3.71	18.7	17.2
12	20.6	17.8 ± 2.29	19.7	17.7
13	21.6	Hi*	20.7	21.6
14	22.6	Hi*	21.7	19.4
15	23.6	Hi*	22.7	20.9
16	24.6	Hi*	23.7	22.0
18	26.6	not tested	25.7	23.2
20	28.6	not tested	27.7	25.2

^§^Measurements were performed in triplicate.

*Manufacturers instructions for the StatStripXPress indicate The Lactate Pro will display “Lo” when lactate levels are below 0.3 mmol/L and “Hi” will be displayed when lactate > 20 mmol/L.

A Deming Regression was used to provide an errors in variables regression model accounting for variation in both x and y variables [9]. A Bland Altman plot then provided a visual assessment of the possible relationship between the measurement error and the true value by plotting the difference against the mean [10]. Precision of the estimated limits of agreement were calculated using standard errors and 95% Confidence intervals for the limits of agreement.

## Findings

### Validation of lactate Pro as an accurate measure of amniotic fluid lactate

**Figure 1:**
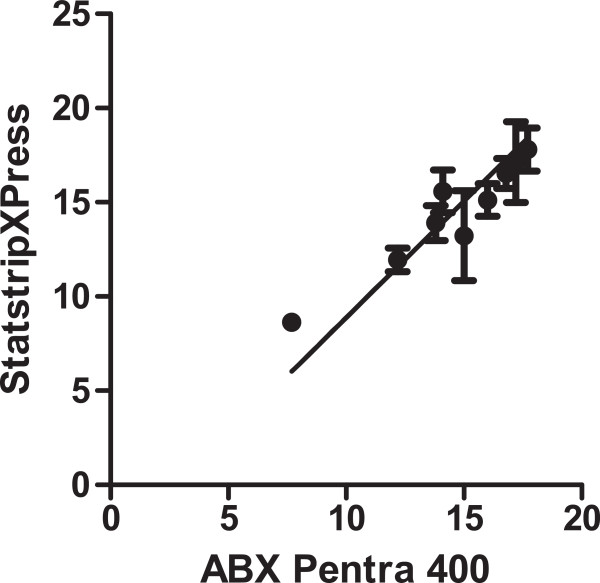
**Deming Regression showing the line of best fit between the concentration of amniotic fluid lactate measured with the StatStripXPress hand held meter and the ABX Pentra 400.**

**Figure 2:**
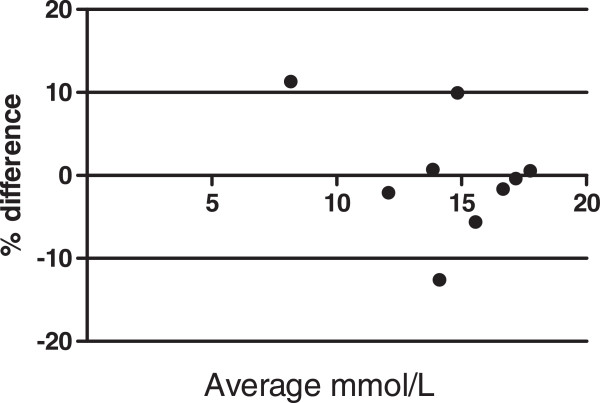
**A Bland- Altman plot showing the agreement between the held meter StatstripXPress and the ABX Pentra 400.** The mean difference between hand held meter and lab instrument was -1.1 mmol/L (SD 3.0 mmol/L) across the range 7.7 mmol/L to 20 mmol/L.

A Deming linear regression showed that there was minimal variation between the two methods with Deming regression equation of ABX Pentra 400 lactate =0.63 (+/-3.7) +0.91*StatStripXPress (+/-0.21) mmol/L (Figure 1).The individual point agreement across the range of test values was determined using the Bland Altman plot. It showed that the average bias of paired samples was -1.0 mmol/L of agreement in the individual point mean difference across the range of lactate values from 7.7 mmol/L to 25 mmol/L, small enough for us to be confident that the hand held method can be used in the clinical setting (Figure 2).

## Discussion

After considering the estimation of the difference in results between the hand held meter and the Pentra 400 using the Bland Altman to plot the difference between the measurements (for each value against their mean) we conclude that results obtained using the hand held method are not sufficiently different to cause problems in clinical interpretation of the lactate concentration in amniotic fluid. The results show the handheld StatStripXPress meter to be reliable measuring lactate in amniotic fluid samples collected and tested in controlled conditions.

Our findings are limited by the use of a single sample of amniotic fluid as the diluent however this limitation reflects the ethical concerns and practical difficulties of obtaining amniotic fluid from pregnant, non-labouring women. In our single sample, the biological baseline lactate level was 7.7 mmol/L and therefore the assay did not capture lactate results below this level. Nonetheless, the performance of handheld lactate meters have been found reliable in a number of clinical studies in which lactate levels are frequently below 7 mmoL in blood [11, 12]. Although a larger sample size may be useful to determine a range for baseline levels of amniotic fluid lactate in non-labouring women and baseline lactate levels at various gestations, this was not the aim of this study.

We remain interested in exploring the use of handheld meters to test amniotic fluid lactate for the reasons of utility in bed side diagnosis, cost efficiency and potential use in remote settings. In future, if a measure of lactate is found to be relevant to the management of labour, lactate tests will need to be easily and reliably measured at the bedside and the role of potential confounders understood.

## Conclusions

This small validation study establishes proof of principle that the StatStripXPress meter, a commercially available product for measuring lactate in whole blood, is a reliable measure of lactate in amniotic fluid collected and tested in controlled conditions.

## Abbreviations

mmol/L: Millimoles/liter
mg/dL: Milligrams/deciliter.
